# Deep learning to enable color vision in the dark

**DOI:** 10.1371/journal.pone.0265185

**Published:** 2022-04-06

**Authors:** Andrew W. Browne, Ekaterina Deyneka, Francesco Ceccarelli, Josiah K. To, Siwei Chen, Jianing Tang, Anderson N. Vu, Pierre F. Baldi

**Affiliations:** 1 Gavin Herbert Eye Institute, Center for Translational Vision Research, Department of Ophthalmology, University of California-Irvine, Irvine, CA, United States of America; 2 Institute for Clinical and Translational Sciences, University of California-Irvine, Irvine, CA, United States of America; 3 Department of Biomedical Engineering, University of California-Irvine, Irvine, CA, United States of America; 4 Department of Computer Science, University of California, Irvine, CA, United States of America; 5 Institute for Genomics and Bioinformatics, University of California, Irvine, CA, United States of America; University of Florida, UNITED STATES

## Abstract

Humans perceive light in the visible spectrum (400-700 nm). Some night vision systems use infrared light that is not perceptible to humans and the images rendered are transposed to a digital display presenting a monochromatic image in the visible spectrum. We sought to develop an imaging algorithm powered by optimized deep learning architectures whereby infrared spectral illumination of a scene could be used to predict a visible spectrum rendering of the scene as if it were perceived by a human with visible spectrum light. This would make it possible to digitally render a visible spectrum scene to humans when they are otherwise in complete “darkness” and only illuminated with infrared light. To achieve this goal, we used a monochromatic camera sensitive to visible and near infrared light to acquire an image dataset of printed images of faces under multispectral illumination spanning standard visible red (604 nm), green (529 nm) and blue (447 nm) as well as infrared wavelengths (718, 777, and 807 nm). We then optimized a convolutional neural network with a U-Net-like architecture to predict visible spectrum images from only near-infrared images. This study serves as a first step towards predicting human visible spectrum scenes from imperceptible near-infrared illumination. Further work can profoundly contribute to a variety of applications including night vision and studies of biological samples sensitive to visible light.

## Introduction

**Fig 1:**
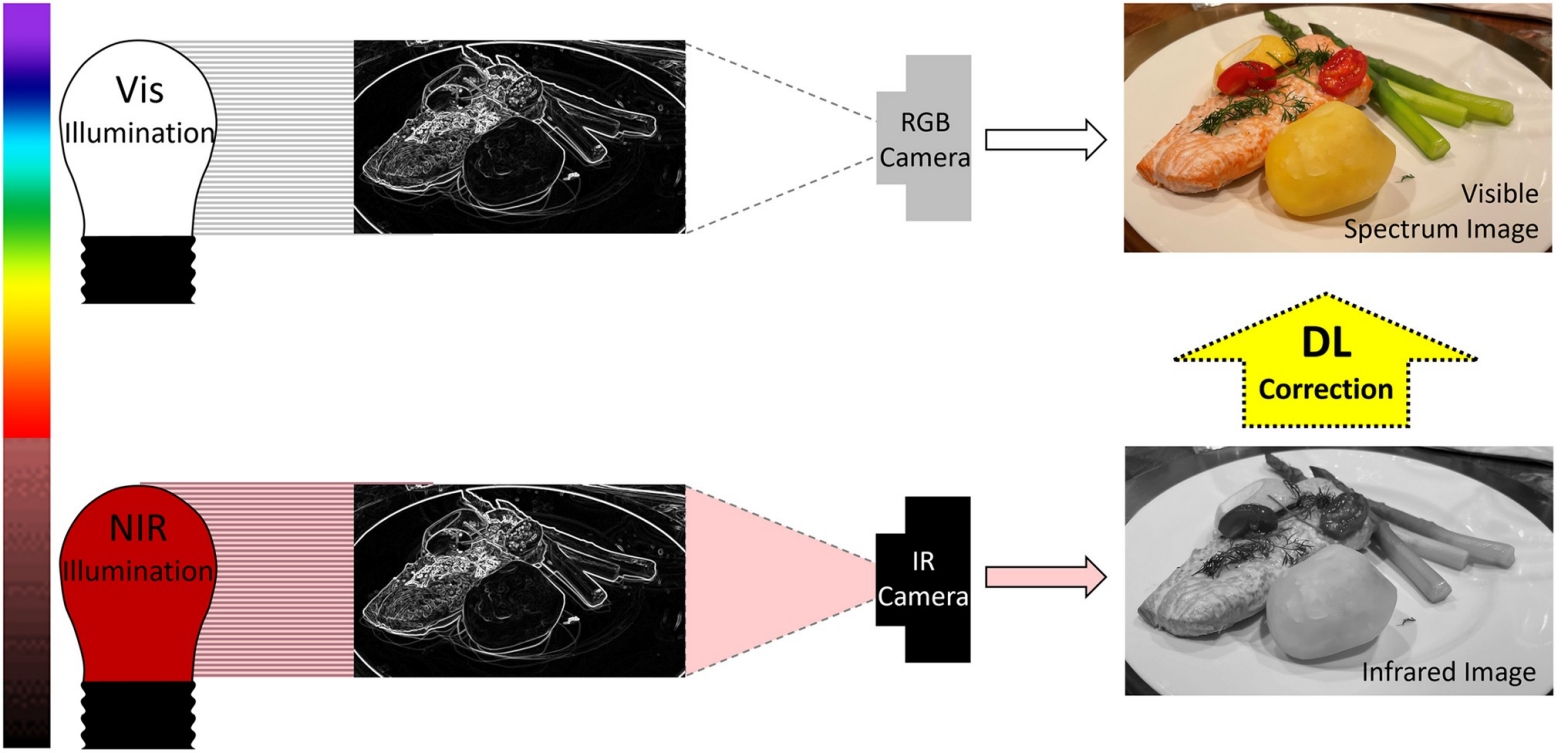
Image processing goal. Predict visible spectrum images using infrared illumination alone and deep learning to process NIR data.

The human eye perceives light in the visible spectrum using opsin proteins bound to a light-sensitive chromophore in retinal photoreceptors. The peak spectral opsin absorbances with surrounding Gaussian distributions for rods, S-cones, M-cones, and L-cones are: 498 nm, 420 nm, 534 nm, and 564 nm respectively [1]. Cone photoreceptors perceive color and are responsible for high acuity vision with rapid phototransduction kinetics. Rod photoreceptors are sensitive to low light conditions offering night vision, but with phototransduction kinetics an order of magnitude slower than cones [2]. Therefore, cones enable the daytime vision of quickly or slowly changing visual scenes, and rods provide night vision but with lower temporal resolution. The peak spectral absorbance for each opsin class defines what is termed the visible spectrum because humans vision occurs in the range of 400–700 nm. Humans do not perceive scenes outside the visible spectrum. In this work, we sought to evaluate the ability of deep learning (DL) [3] coupled with infrared spectroscopy to render visible spectrum images using infrared light illumination and no visible spectrum light. Night vision systems seek to illuminate the environment with infrared (IR) light that is detected and visualized by conventional camera sensors and render a scene in the visible spectrum on a digital display. Historically, night vision systems render scenes as a monochromatic green display. Newer night vision systems use ultrasensitive cameras to detect and amplify visible light. Computer vision tasks with low illuminance imaging have employed image enhancement and deep learning to aid in object detection and characterization from IR spectrum, but not with accurate interpretation of the same scene in the visible spectrum [4]. Conventional cameras acquire blue (B), green (G), or red (R) pixels of data to produce a color image perceptible to the human eye (Fig 1 top row). We investigated if a combination of infrared illuminants in the red and near-infrared (NIR) spectrum could be processed using deep learning to recompose an image with the same appearance as if it were visualized with visible spectrum light (Fig 1 bottom row). We established a controlled visual context with limited pigments to test our hypothesis that DL can render human-visible scenes using NIR illumination that is, otherwise, invisible to the human eye.

Modern color printers produce color images using 4 ink colors: cyan (C), magenta (M), yellow (Y), and black (K). Under broad visible light and human perception, each of these ink colors appears cyan, magenta, yellow or black based on the incident light that they reflect. Spectral reflectance is the percent of light energy that a compound reflects for each specific wavelength of illuminating light. Ink dye’s spectral reflectance is determined by systematically illuminating the dyes across a range of the electromagnetic spectrum and measuring the reflected light. Therefore, printed images from a CMYK printer provide a simplified context to test our hypothesis that NIR illuminated images can be processed to render visible spectrum images that match the images illuminated with visible light.

## Related works

The NIR image colorization problem shares some similarities with the general image colorization and color transfer problems [5–7] with several significant distinctions. In grayscale-to-RGB reconstruction, luminance, i.e. the intensity of light emitted from a surface, can be directly obtained from the grayscale images so only the chrominance needs to be estimated. This greatly simplifies the reconstruction task. Moreover, in color transfer problems, the input and output are both three-channel RGB images, whereas NIR images have only a single channel making the color reconstruction more challenging. Early image colorization techniques required human input to correctly identify similar neighborhood intensity values and assign them a similar color, i.e Scribble [8] or Similar images [9, 10]. Other approaches [11–13] automatically matched patches of the input image to patches of reference image via feature extraction and matching. Despite being fully automatic, these methods require a fitting reference image database and retrieval mechanism to be implemented. More recent approaches [14, 15] leveraged deep neural networks to propose a single framework where image colorization can be learned end-to-end.

Other groups have explored colorizing IR images by several approaches. Toet employed a look-up-table approach where first order statistics were used to develop a color transfer method to evaluate luminance distributions in monochromatic images (such as from Infrared cameras) and assign chromaticity values to each pixel [16, 17]. Liu et al. applied two stages of DL to first apply textural details to a thermal image acquired using a single IR camera. They subsequently added color information to the texturalized thermal image [18]. Zheng et al. studied qualitative and quantitative metrics for night vision colorization and identified computationally rapid colorization by a combination of histogram matching and statistical matching between source IR image and a target color image [19]. They identified trade-offs in contrast and saturation at the expense of computational time. Suarez et al. employed Deep Convolutional Generative Adversarial Network to colorize single-channel near IR images from city scenery and succeeded in colorization at the expense of image clarity [20]. Mehri et al. proposed a Cycle-Consistent adversarial network to predict color channels from a single NIR input on unpaired dataset [21]. Each of these infrared to color image techniques rely on single IR image input for images acquired in the real world. Brown and Susstrunk demonstrated improved scene category recognition when color images of scenes were supplemented by a NIR image channel [22]. We, therefore, sought to systematically study infrared colorization for multiple infrared wavelengths in a controlled imaging context.

Deep neural networks [3], particularly Convolutional Neural Networks (CNNs), have achieved state-of-the-art performances in a variety of computer vision problems. CNNs, originally inspired by the seminal work of Hubel and Wiesel [23], were developed by Fukushima [24], using however the wrong learning algorithm [25]. They were perfected in the 1980s-1990s using the backpropagation learning algorithm [26, 27] and are used today across most computer vision tasks [28, 29], including biomedical imaging tasks, with thousands of references in the literature (e.g. [30–34] from our group alone). Recognition CNNs convolve the input image through a hierarchical series of adaptive filters that are learned from the data and produce increasingly more abstract representations of the data supporting translation invariant recognition. Generative CNNs operate in the reverse direction and both recognition and generation networks can be combined in various architectures. In particular, in this work we use architectures inspired by the U-Net architecture [35] in which a contracting path is combined with an expansive path (yielding a “U” shape architecture). The U-Net architecture has been used effectively in several tasks, from image segmentation to photoacoustic imaging reconstruction [35–37].

## Materials and methods

### Multispectral image acquisition

**Fig 2:**
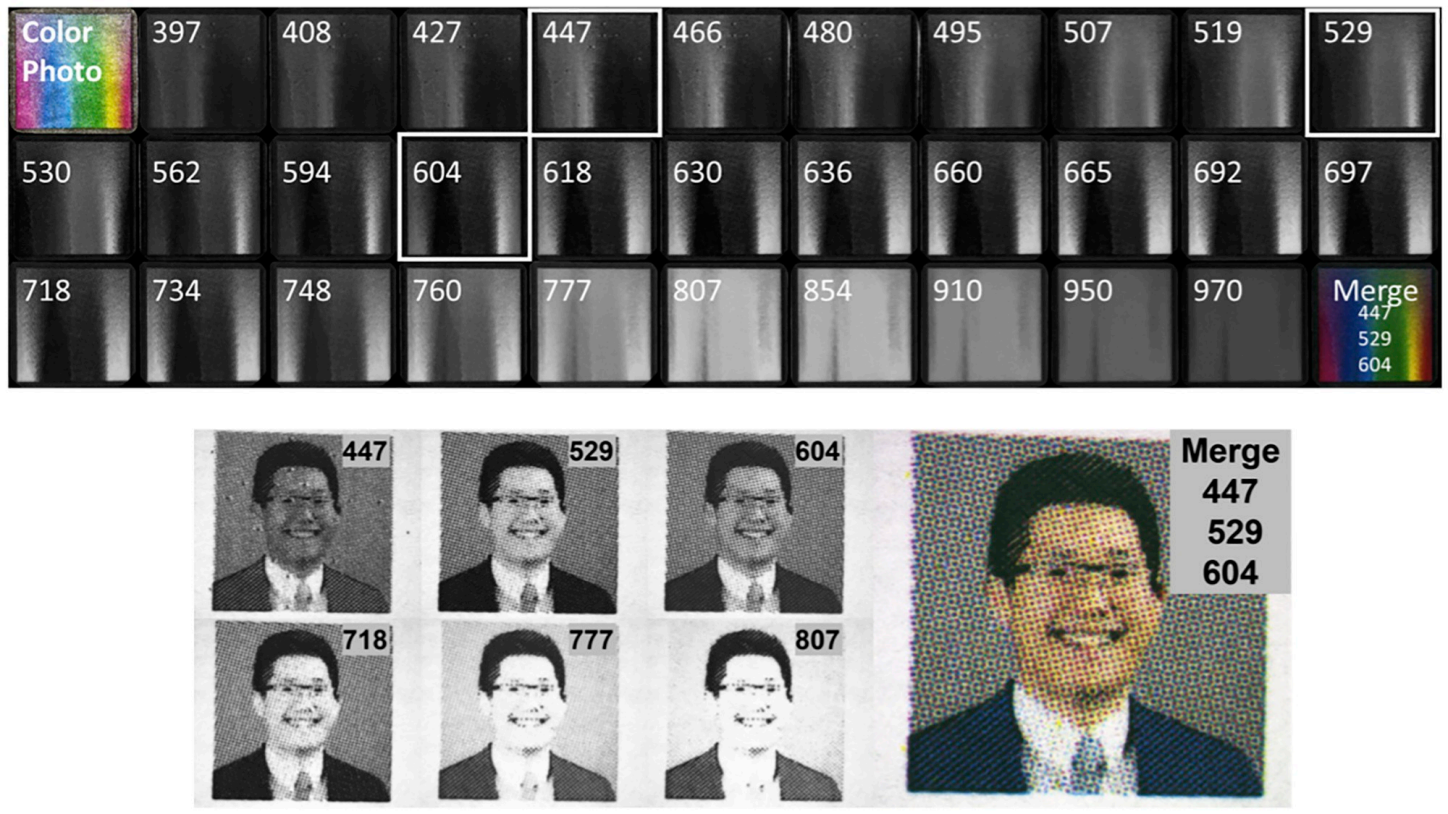
Sample images from the human portraits library. *(top row)* Spectral reflectance across 32 channels of printed a Windows color palette with color photo and merged channels 447, 529, 604. *(bottom row)* Spectral reflectance for 6 selected illuminant wavelengths and the visible spectrum color a photo created by merging channels 447, 529, 604.

To learn the spectral reflectance for cyan, magenta and yellow ink, we printed a rainboy color pallete using a using a Canon office printer with CMYK ink. Examples of the color palette under multispectral illumination (center wavelengths: 397, 408, 427, 447, 466, 480, 495, 507, 519, 529, 530, 562, 594, 604, 618, 630, 636, 660, 665, 692, 697, 718, 734, 748, 760, 777, 807, 854, 910, 950, 971 nm) is shown in Fig 2. Photographs of each printed image under multispectral illumination were acquired using a monochromatic camera (Zeiss Axiocam 305, CarlZeiss) mounted on a dissection microscope focused on the image. To account for diminishing camera quantum efficiency in the red and infrared spectrum, each image channel was normalized to the white background of the paper upon which the images were printed. For both panels in Fig 2, the right inset is an image combined by assigning the 447 nm, 529 nm, and 604 nm images to the red, green, and blue channels respectively. Channel assignment and merging were performed in imageJ [38]. Spectral reflectance was determined for each C, M, Y printer dye using a normalized plot profile function (imageJ) for each of the 31 LED illuminants.

**Fig 3:**
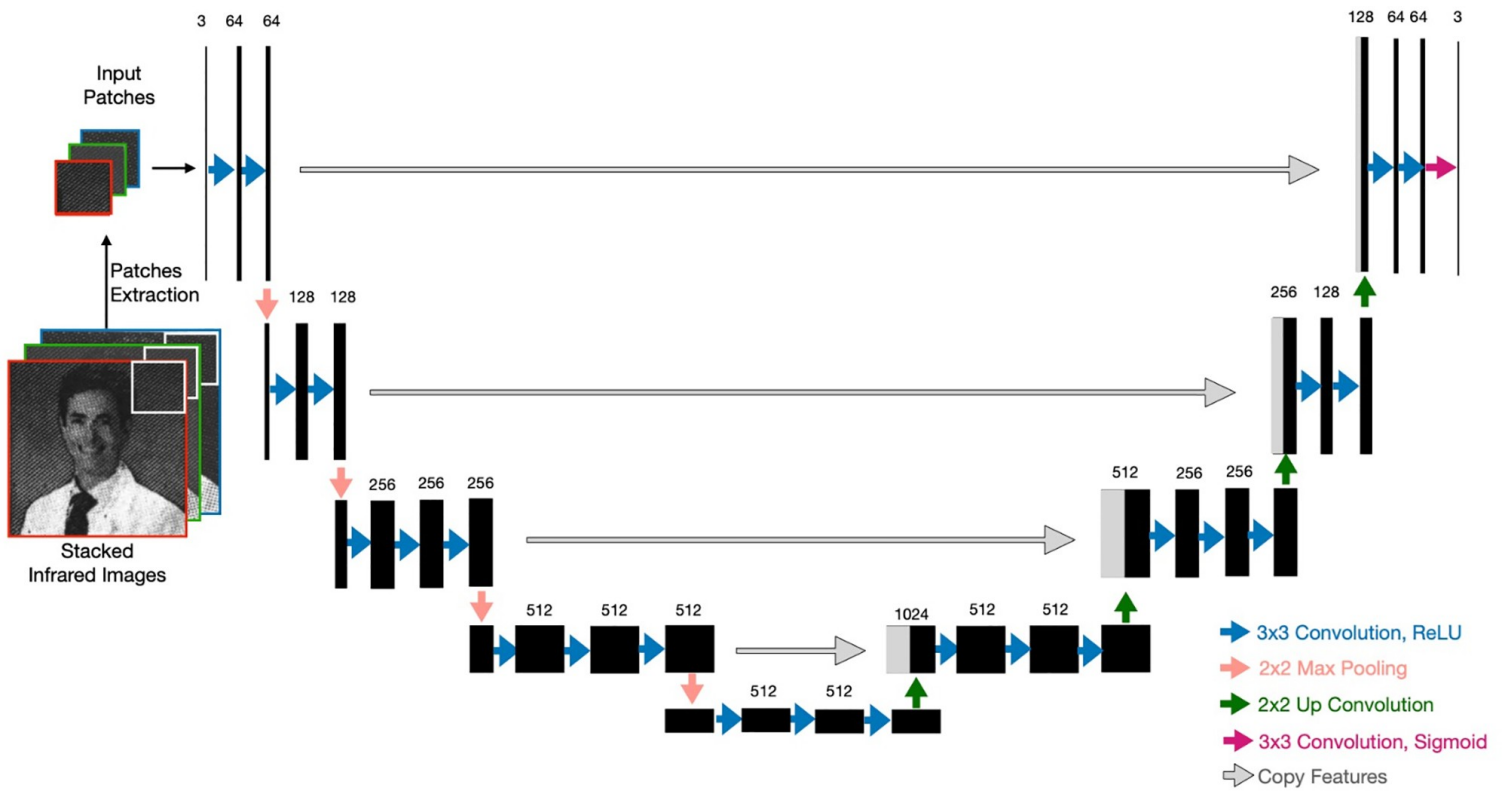
U-Net architecture for infrared colorization. The number of channels is reported on the top of the feature maps. The arrows represent different types of operations.

We printed a library of over 200 human faces available from the publicly available “Labeled Faces in the Wild” repository (http://vis-www.cs.umass.edu/lfw/) [39]. The images were printed using a using a Canon office printer with CMYK ink. We acquired photographs of each image under different wavelengths of illumination to then be used to train machine learning models to predict RGB color images from individual or combinations of single wavelength illuminated images. Each image in the dataset was illuminated with diffuse uniform illumination by 6 different LED wavelengths spanning the visible and near-infrared spectrum (center wavelengths: 447, 529, 604, 718, 777, 807 nm). The datasets are available for downloading (https://dx.doi.org/10.6084/m9.figshare.19291166). The individual pictured in manuscript Figs 2, 3 and 9 provided written informed consent (as outlined in PLOS consent form) to publish their image alongside the manuscript.

### Baseline and network architectures

To predict RGB color images from individual or combinations of wavelength illuminations, we evaluated the performance of the following architectures: a baseline linear regression, a U-Net inspired CNN (UNet), and a U-Net augmented with adversarial loss (UNet-GAN).

#### Linear regression baseline

We implemented a simple linear regression model as a baseline to compare the results of the deep neural architectures and assess their performances. As input to the linear model, we evaluated patches of several sizes and predicted target patches separately for each color channel (R, G, and B).

#### Conventional U-Net

A traditional U-Net is a fully convolutional network that consists of a down-sampling path (encoder) that helps to capture the context and an up-sampling path (decoder) that is responsible for precise localization. The down-sampling path (left side of Fig 3) repeatedly applies a series of operations which consists of three 3 × 3 convolutions followed by rectified linear unit (ReLU) activation and a 2 × 2 max pooling operation. At the end of one series of operations, the number of feature maps is doubled and the size of feature maps is halved. The up-sampling path (right side of Fig 3) repeatedly performs another series of operations consisting of an up-convolution operation of the feature maps by a 2 × 2 filter, a concatenation with the corresponding feature map from the down-sampling path, and two 3 × 3 convolutions, each followed by a ReLU activation. In the output layer, a 3 × 3 convolution is performed with a logistic activation function to produce the output. Padding is used to ensure that the size of output matches the size of the input.

#### VGG network as a U-Net encoder (UNet)

VGG neural networks [40], named after the Visual Geometry Group that first developed and trained them, were initially introduced to investigate the effect of the convolutional network depth on its accuracy in large-scale image recognition settings. There are several variants of VGG networks like VGG11, VGG16, and VGG19, consisting of 11, 16, and 19 layers of depth, respectively. VGG networks process input images through a stack of convolutional layers with a fixed filter size of 3 × 3 and a stride of 1. Convolutional layers are alternated with max-pooling filters to down-sample the input representation. In [41], using VGG11 with weights pre-trained on ImageNet as U-Net encoder [42] was reported to improve the performance in binary segmentation. In this study, we tried a U-Net architecture with a VGG16 encoder [40] using both randomly initialized weights and weights pre-trained on ImageNet. We refer to this architecture simply as U-Net.

#### U-Net with adversarial objective (U-Net-GAN)

To enhance the performance of the U-Net architecture, we also explored a Conditional Generative Adversarial Network approach (CGAN). A conventional CGAN consists of two main components: a generator and a discriminator. The task of the generator is to produce an image indistinguishable from a real image and “fool” the discriminator. The task of the discriminator is to distinguish between real and fake images produced by the generator, given the reference input images. In this work, we examined the performances of two U-Net-based architectures: 1) a stand-alone U-Net implementation as described in the previous section and 2) a CGAN architecture with a U-Net generator along with an adversarial discriminator. For the second approach, we used a PatchGAN discriminator as described in [7], which learns to determine whether an image is fake or real by looking at local patches of 70 × 70 pixels, rather than the entire image. This is advantageous because a smaller PatchGAN has fewer parameters, runs faster, and can be applied on arbitrarily large images. We refer to this latter architecture (U-Net generator and PatchGAN discriminator) as U-Net-GAN.

### Experimental settings and training

For all the experiments, following standard machine learning practice, we divided the dataset into 3 parts and reserved 140 images for training, 40 for validation and 20 for testing. To compare performances between different models, we evaluated several common metrics for image reconstruction including Mean Square Error (MSE), Structural Similarity Index Measure (SSIM), Peak Signal-to-Noise Ratio (PSNR), Angular Error (AE), DeltaE and Frechet Inception Distance (FID). FID is a metric that determines how distant real and generated images are in terms of feature vectors calculated using the Inception v3 classification model [43]. Lower FID scores usually indicate higher image quality. FID has been employed in many image generation tasks including NIR colorization study [21]. However, Mehri et al. used a single-channel NIR input whereas we feed three stacked images of different NIR wavelengths. Therefore, we cannot entirely compare FID values between our studies as the settings are different. We report FID in the main manuscript and comprehensively present additional metrics in the Supplementary section.

#### Linear regression baseline

For the linear regression model, the input images were divided into patches. We explored several patch sizes of 6 × 6, 12 × 12, 24 × 24 and 64 × 64 pixels. Every patch from the selected input infrared images was used to predict the corresponding R, G, and B patch of the target image. A different linear model was used for each color channel and the results for one image were computed as the average over the three channels.

#### U-Net and U-Net-GAN

To train the proposed deep architectures, we divided the input images into random patches of size 256 × 256. Random cropping of the patches was used as a data augmentation technique. Both inputs and outputs were normalized to [−1, 1]. The deep architectures were trained for 100000 iterations with a learning rate starting at 1 × 10^−4^ and cosine learning decay. Given the fully convolutional nature of the proposed architectures, the entire images of size 2048 × 2048 were fed for prediction at inference time. As a loss function for neural networks, i.e. U-Net and U-Net-GAN, we used mean absolute error (MAE).

#### Human grader evaluation of model performance

We performed a human evaluation of model performance. We randomly sampled 10 images (ground truth and the 3 prediction output by UNet-GAN, UNet and Linear regression). We provided a blinded multiple choice test to 5 graders to select which of the 3 outputs subjectively appeared most similar to the ground truth image. We subsequently randomly selected 10 patches (portion of images) and asked graders to compare similarity of predicted patches from UNet-GAN and UNet with the corresponding ground truth patch.

## Results

In this section, we first describe our spectral reflectance findings for CMY printed inks imaged under different illuminant wavelengths and then report and discuss the performances for the different architectures to accurately predict RGB images from input images acquired by single and combinations of different wavelength illumination.

### CMY spectral reflectance

To identify the range of wavelength with the most distinct reflectance profile, we conducted CMY spectral reflectance analysis. Inluminant wavelengths with spectral reflectances that are unique for each ink color were assumed to contain the most valuable information for color reconstruction.

**Fig 4:**
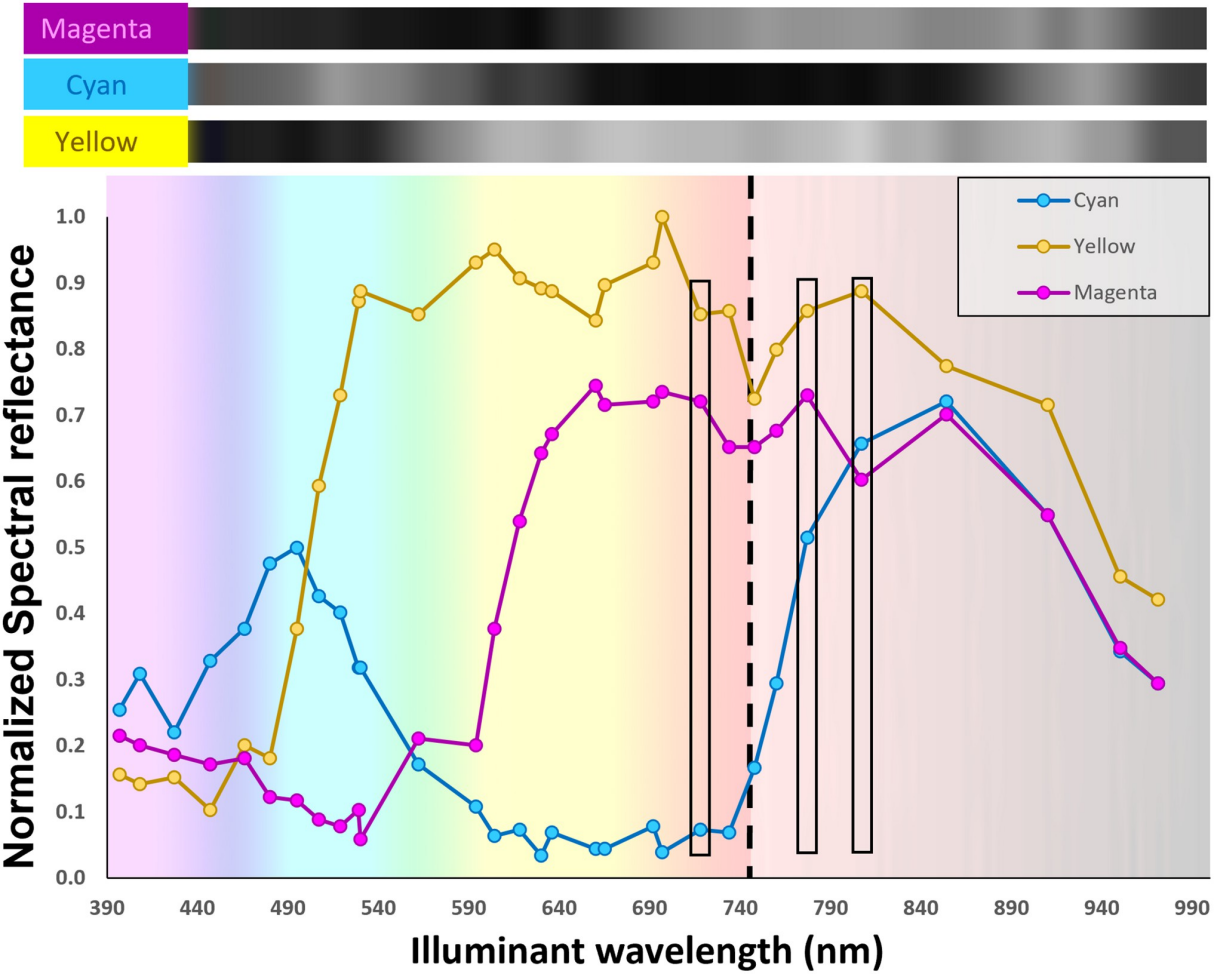
Spectral reflection analysis of cyan, magenta, and yellow inks illuminated with 31 LED channels spanning visible to near-infrared illumination. Images of each dye were captured by a monochromatic camera and grayscale reflectance intensity sequentially arrayed in the top inset.

Spectral reflectance of cyan, magenta, and yellow inks was determined by multispectral illumination and plotted according to the detected grayscale value (Fig 4). The top inset demonstrates the grayscale reflectance values for each ink with ascending wavelength of illumination (total of 31 wavelengths of illumination). The plot in Fig 4 is the normalized quantified reflectance across visible and NIR spectra. Cyan and magenta inks have a peak reflectance with the 495 and 660 nm LED illuminants respectively. Yellow ink had a broader reflectance with peak reflectance for 604 and 697 nm illuminants. Cyan ink demonstrated an isolated reflectance maximum in the NIR spectrum around 854 nm, while yellow and magenta inks demonstrated broad reflectance in the NIR spectrum. Spectral reflectance profiles were distinct for each ink color, but cyan and magenta demonstrated overlapping reflectance profiles above 854 nm. Spectral reflectance demonstrated no overlap for 718, 777 and 807nm (black box outlines in Fig 4).

### Infrared to RGB reconstruction

#### RGB reconstruction

**Fig 5:**
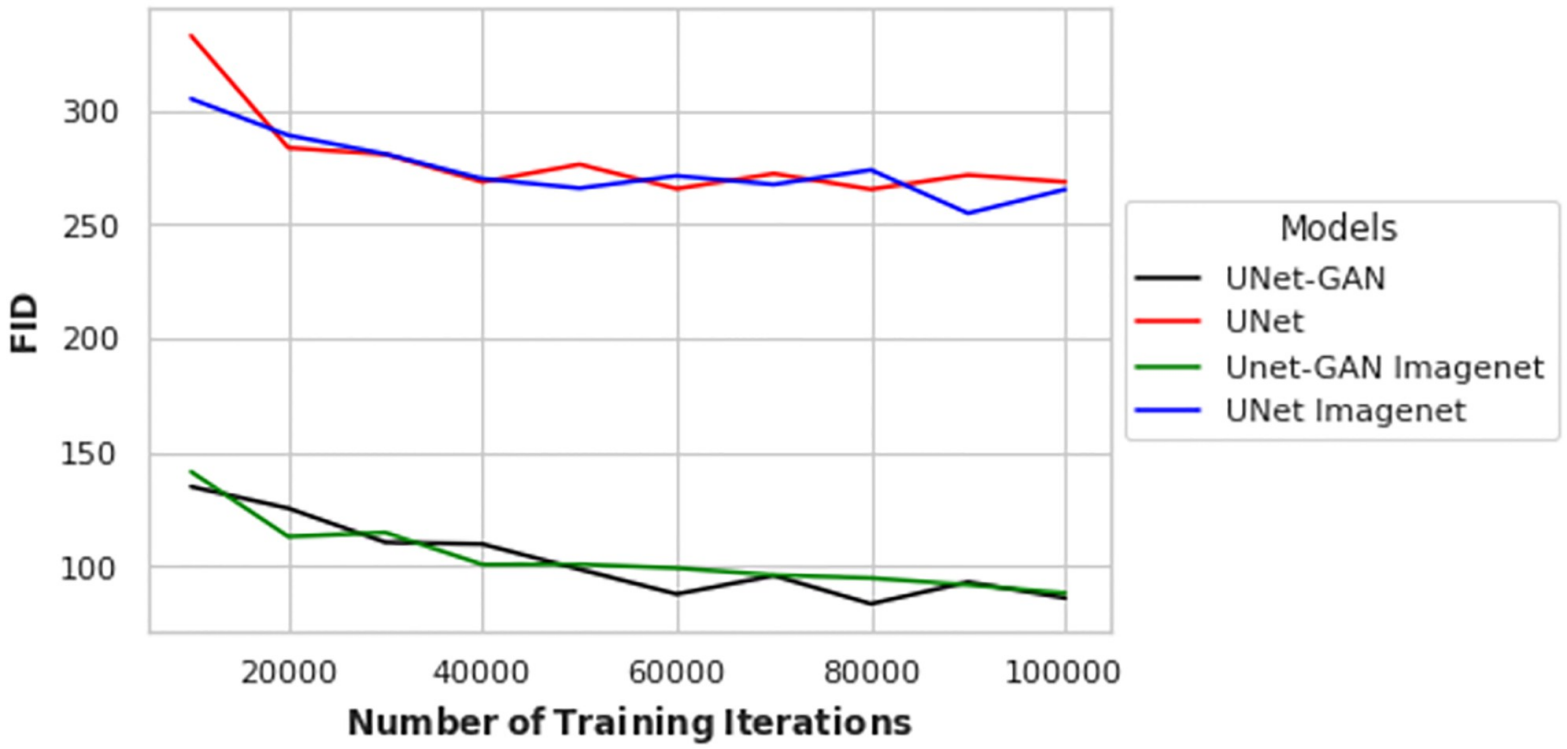
Reconstruction performances for the UNet-based architectures. FID scores and number of iterations for the UNet-based architectures.

**Fig 6:**
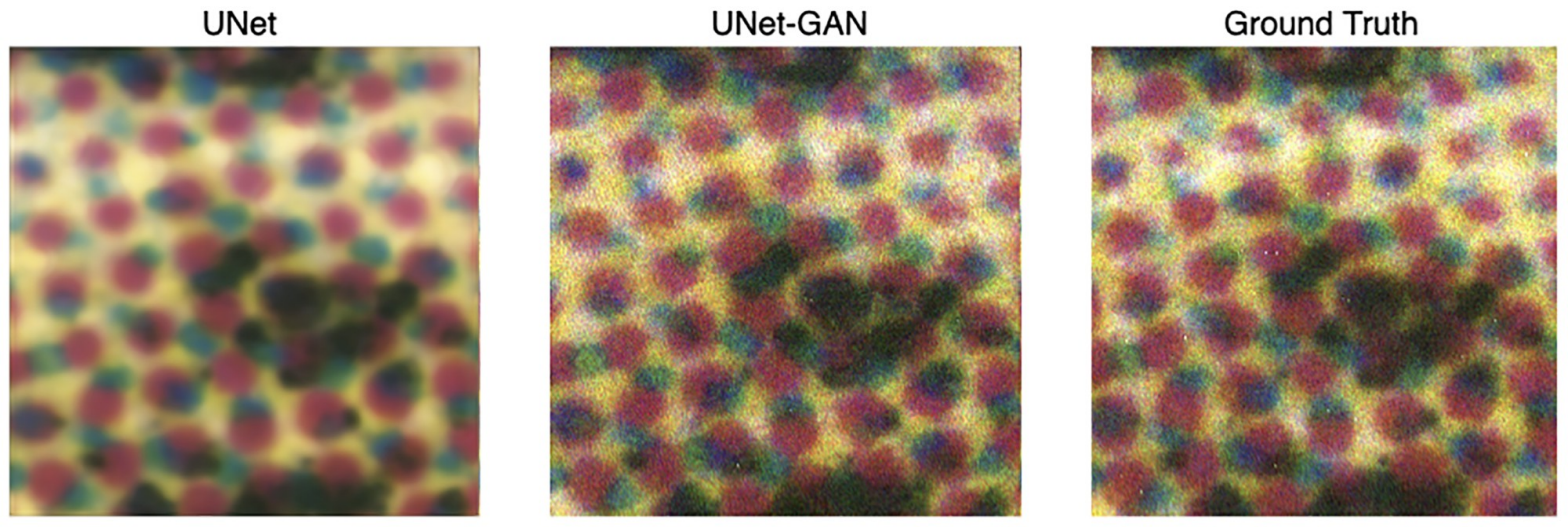
Visualization of the reconstructed RGB patches. *(left)* Patch generated by the UNet architecture. *(middle)* Patch generated by the UNet architecture with adverserial loss. *(right)* Ground truth RGB patch.

Since transfer learning has been widely used to improve deep model performances and reduce the risk of overfitting, we explored four slight variations of the main learning architectures. That is, using the three selected wavelengths (718, 777, and 807), we evaluated a UNet-GAN and a UNet architecture, both with or without ImageNet pretrained weights. Quantitative results are shown in Fig 5, where the *x*-axis denotes the number of iterations and the *y*-axis the FID scores. From Fig 5, it is observed that the adverserial objective enables predictions to reach lower FID scores. Furthermore, the use of ImageNet pretraining (Fig 5 does not boost the performances, leading in some cases to higher FID scores. A possible explanation could be the difference in the domain of the ImageNet dataset, which does not contain a lot of human images, while human portraits are used in the current study. The experimentation that followed did not include ImageNet pretrained weights. To further qualitatively assess the difference in performances and to validate the use of the FID as a metric reflective of human perception, Fig 6 shows the reconstruction of a ground truth RGB patch (*right*) by UNet (*left*) and UNet-GAN (*middle*). From visual inspection of Fig 6, it is evident that adopting adverserial loss results in significantly sharper reconstructions which are much closer to the ground truth both in terms of colors and patterns.

**Fig 7:**
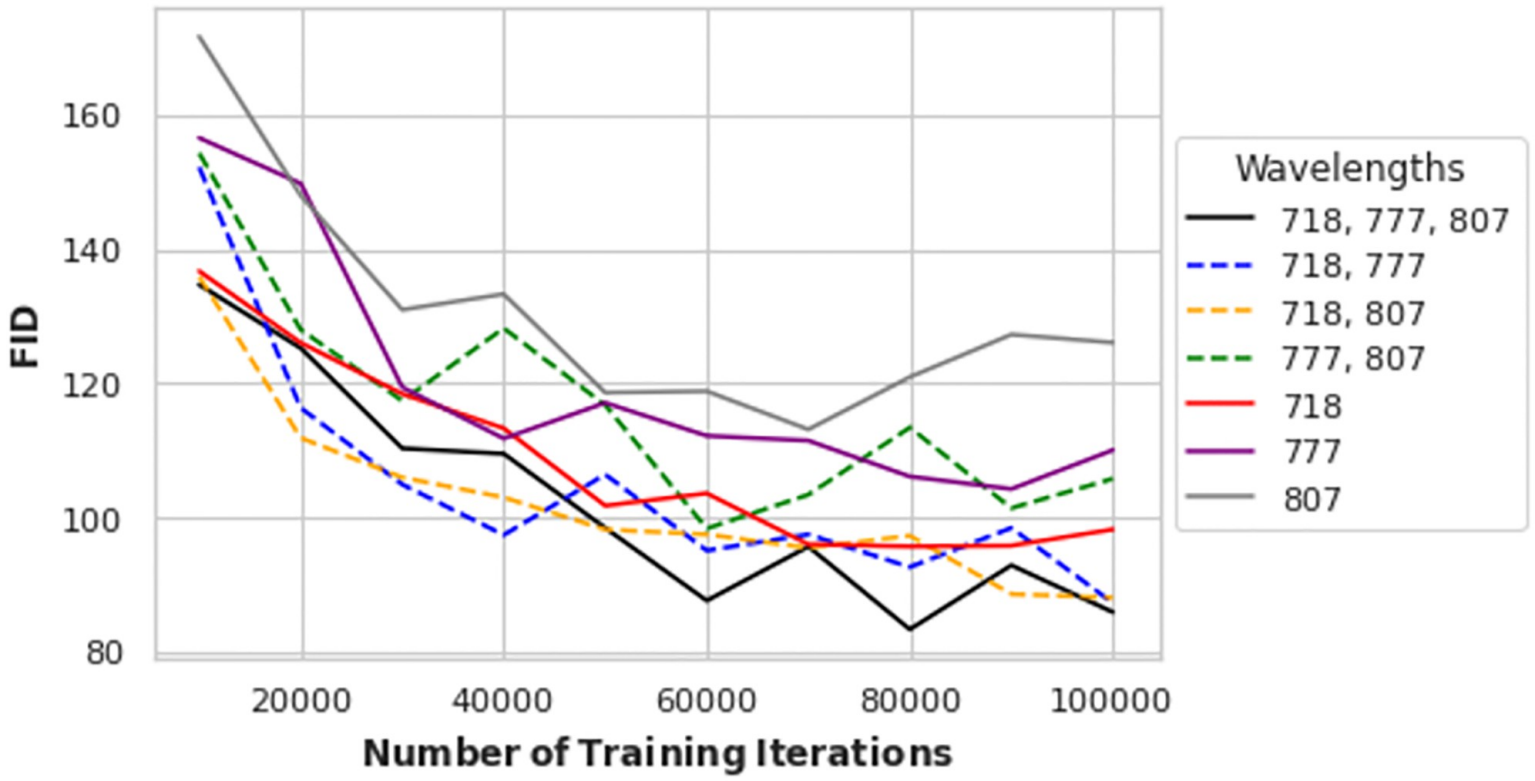
Reconstruction performances with several input illuminations. FID scores and number of iterations for the UNet-based architectures using different combinations of input wavelengths.

To determine if three input wavelengths (718, 777, and 807) provided benefit over single or doublet wavelength inputs, we evaluated the UNet-GAN architecture using all the possible combinations of two wavelengths, three wavelengths, and single infrared illuminations as inputs. The quantitative results of these experiments are shown in Fig 7. From Fig 7, it appears that using single infrared illuminations generally leads to poor reconstructions, while combining two infrared inputs, like 718 and 777 or 718 and 807, is useful to improve performances. The use of the three selected wavelengths results in the lowest FID score, which is reached at 80000 iterations. For further evaluations we used the models at 80000 iterations.

**Fig 8:**
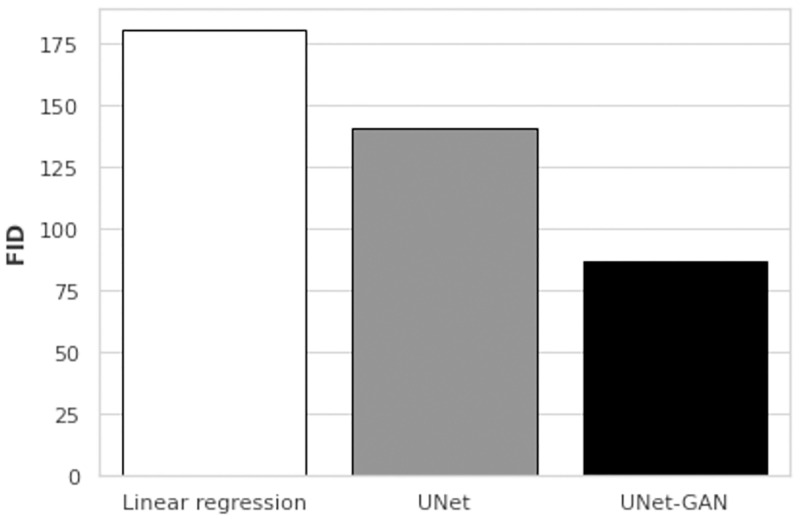
Models comparison with a regression baseline. FID scores for the baseline linear regression (FID score: 180.5), UNet (FID score: 140.8) and UNet-GAN (FID score: 87.2) on the same test set.

**Fig 9:**
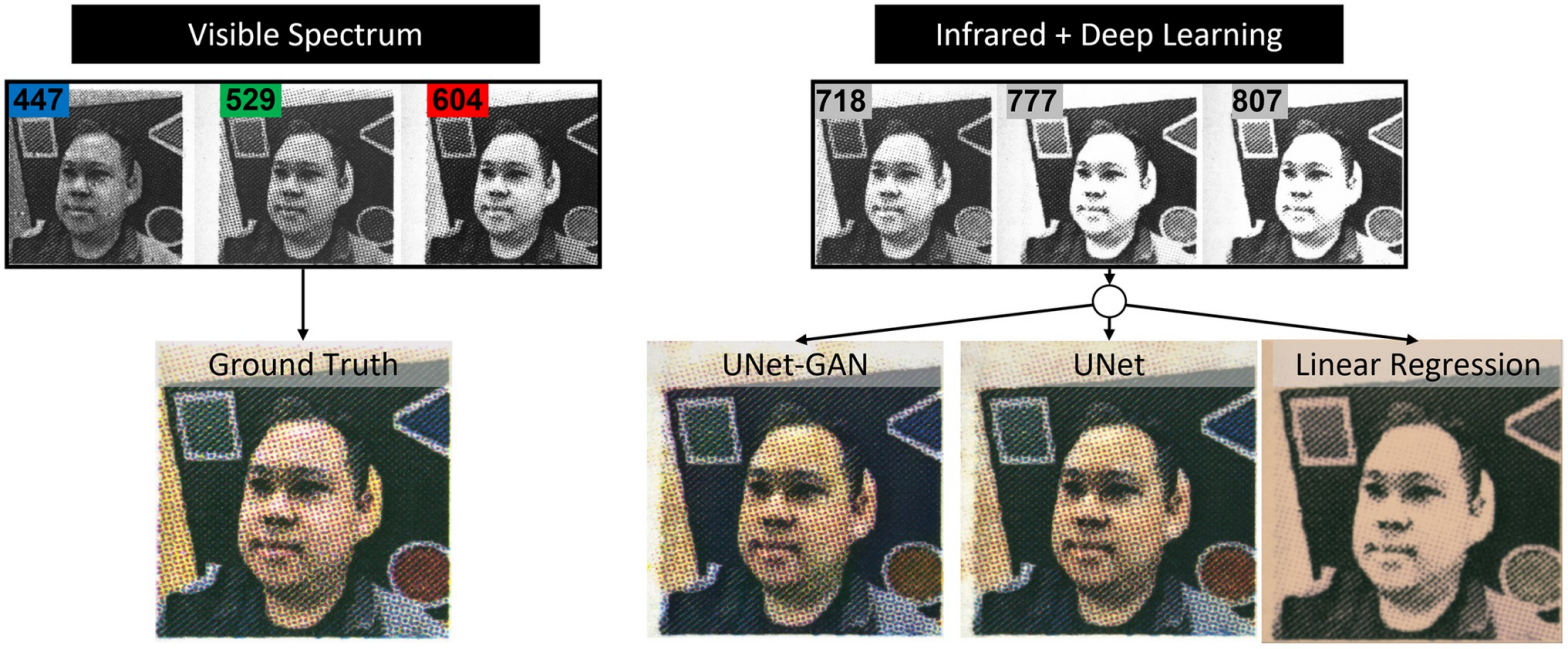
Visualization of the reconstructed RGB images by the deep architectures and baseline regression model. (*left*) Visible spectrum ground truth image composed of red, green and blue input images. (*right*) Predicted reconstructions for UNet-GAN, UNet and linear regression using 3 infrared input images.

To compare the deep architectures with a baseline, we assessed the performances of UNet, UNet-GAN at 80000^th^ iterations and a linear regression model on the same test set composed of 20 images. The quantitative results are shown in Fig 8. The simple linear regression baseline failed to accurately reconstruct the ground truth RGB images and results in the highest FID scores. Furthermore, as qualitatively presented in Fig 6, the use of adversarial loss allows for sharper reconstructions and results in a much lower FID score (140.8 vs 87.2). Qualitative comparison of RGB reconstructions are presented in Fig 9. Simple linear regression model produces images lacking color information seen in ground truth images and in the images produced by UNet and UNet-GAN architectures. While the UNet and UNet-GAN reconstructions appear similar upon gross inspection, the patch analysis shown in Fig 6 demonstrates the qualitatively closer match between the adversarial network and ground truth than the non-adversarial network and ground truth. The qualitative observations are mirrored by the lower FID score of 87.2 of the adversarial network (Fig 8).

Finally, human subject comparison of model outputs with ground truth inputs was used to determine perceived model performance. In all cases, the graders determined that linear regression had worse performance than UNet-GAN or UNet. In 52% of cases, human graders determined that UNet outperformed UNet-GAN. However, when zooming into predicted images at the patch level, UNet-GAN was perceived to out-perform UNet 100% of the time. UNet patch predictions, while similar in color content, were more blurry than UNet-GAN patches.

## Discussion and conclusion

We have investigated the reconstruction of RGB images, given their representation under infrared illuminations, using deep neural networks. We have evaluated two slight variations of deep architectures along with a linear regression baseline model and different weights initialization techniques using an in-house generated dataset. The deep U-Net-based architectures proved able to generate visually consistent RGB reconstructions using only three input infrared images. These reconstructions were significantly better than the ones produced by the linear model, both quantitatively and qualitatively through visual inspection. The use of adversarial training was useful to produce significant improvements, often resulting in lower FID scores. Human subject evaluation consistently determined that UNet and UNet-GAN models outperformed linear regression, and UNet moderately outperformed UNet-GAN at colorizing infrared images. This demonstrates the limitations of objective computational metrics, like FID, to fully describe human perception.

This work suggests that predicting high-resolution images is more dependent upon training context than on spectroscopic signatures for each ink. In this work, four compounds (C, M, Y, K inks) were characterized spectroscopically using a monochromatic camera sensor in the contexts of rainbow input images and naturally occurring images. One might hypothesize that larger separation of in spectral reflectance curves for each ink would aid CNNs to predict a color image with lower error because curve overlap could introduce represent spectroscopically indistinguishable information. For example, spectral reflectance curves for CMY inks at 718 and 777 nm illumination demonstrate significantly different curve separations (Fig 4). For 718 nm illumination, the reflectance of cyan, magenta, and yellow are 0.1, 0.7, and 0.9 respectively. For the 777 nm illuminant, the spectral reflectances are much closer in value with cyan, magenta, and yellow inks having normalized reflectances of 0.5, 0.7, and 0.9 respectively.

Another important aspect we aimed to address was the speed of the predictions. To enable real-time color vision in complete darkness for video data, the inference time should be small enough so that a sufficient number of frames can be processed each second. The proposed U-Net architectures are capable of producing three images per second, without using any particular accelerations. We tried several generator backbones and reported the inference times in the Supplementary. This provides a starting point for further speed improvements that could use, for instance, smaller architectures, or multi-threading, or better hardware.

This proof-of-principle study using printed images with a limited optical pigment context supports the notion that the deep learning approach could be refined and deployed in more practical scenarios. These scenarios include vision applications where little visible light is present either by necessity or by goal. Night vision, for example, has applications in security, military operations, and animal observation. Similarly, handling, processing, and studying samples sensitive to visible light may require a technology that uses no visible light. Studying the light-sensitive retinal tissue, for example, may require processing the sample in darkness to avoid altering its biochemistry and function [44, 45]. Likewise, performing eye surgery benefits from low light exposure to avoid retinal damage [46]. Similarly, handling restoration of light-sensitive artifacts may benefit from minimizing exposure to visible light.

In short, this study suggests that CNNs are capable of producing color reconstructions starting from infrared-illuminated images, taken at different infrared wavelengths invisible to humans. Thus, it supports the impetus to develop infrared visualization systems to aid in a variety of applications where visible light is absent or not suitable.

## Supporting information

S1 File (PDF)
