# Supplementary material for: Deep learning to enable color vision in the dark
Source: PLoS One. 2022 Apr 6;17(4):e0265185. doi: 10.1371/journal.pone.0265185 (PMC8985995; doi:10.1371/journal.pone.0265185)
Supplement: S1 File — (PDF) [file pone.0265185.s001.pdf]

Deep Learning to Enable Color Vision in the Dark

Andrew W. Browne[1,2,3*†], Ekaterina Deyneka[4,5†], Francesco Ceccarelli[4,5†], Siwei Chen[4,5], Josiah K. To[1], Jianing Tang[3], Anderson N. Vu[1], Pierre Baldi[4,5*]

1 Gavin Herbert Eye Institute, Department of Ophthalmology, University of California-Irvine, Irvine, CA 92617, USA
2 Institute for Clinical and Translational Sciences, University of California-Irvine, Irvine, CA 92617, USA
3 Department of Biomedical Engineering, University of California-Irvine, Irvine, CA 92617, USA
4 Department of Computer Science, University of California, Irvine, CA, USA 92627
5 Institute for Genomics and Bioinformatics, University of California, Irvine, CA, USA 92627

†These authors contributed equally
* Corresponding authors: abrowne1@uci.edu, pfbaldi@uci.edu

Supplementary

Training details and models evaluation

For all the experiments, we divided the dataset into 3 parts and reserved 140 images for training, 40 for validation and 20 for testing. To compare performances between different models, we evaluated several common metrics for image reconstruction including Mean Square Error (MSE), Structural Similarity Index Measure (SSIM), Peak Signal-to-Noise Ratio (PSNR), Angular Error (AE), DeltaE and Frechet Inception Distance (FID). FID is a metric that determines how distant real and generated images are in terms of feature vectors calculated using the Inception v3 classification model [1]. Lower FID scores usually indicate higher image quality.

We aimed at selecting the metric that reflects human perception the best for our task. For this, we calculated all the above-mentioned scores and visually inspected the results. All models in our experiments were trained for 100000 iterations with a learning rate starting at 1×10^{-4} and cosine learning decay using randomly cropped patches of the size 256×256 and normalization to $[-1, 1]$. Given the fully convolutional nature of the proposed architectures, the entire images of size 2048×2048 were fed for prediction at inference time. As a loss function for neural networks, i.e. U-Net and U-Net-GAN generator, we used mean absolute error (MAE).

In the next sections we will describe the experimental settings and provide the graphs with metrics, which helped us to identify the best image evaluation metric and best model.

Model selection

To reconstruct RGB images from individual or combinations of near-infrared illumination we tried the following four modifications of UNet-like models: U-Net inspired CNN, U-Net inspired CNN with ImageNet pretrained weights, U-Net augmented with adversarial loss (model similar to Pix2Pix [2]), U-Net augmented with adversarial loss with ImageNet pretrained weights.

The following graphs were obtained using validation dataset.

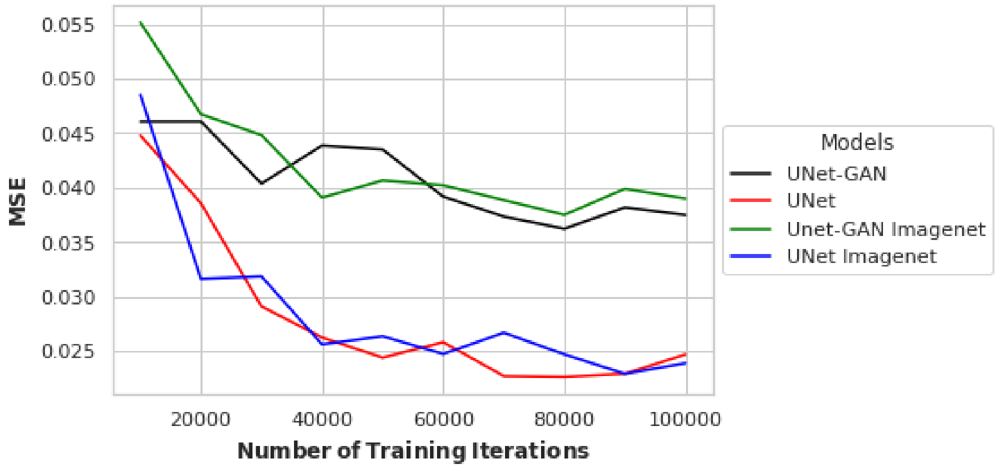

Fig 1. MSE scores for four reconstruction pipelines.

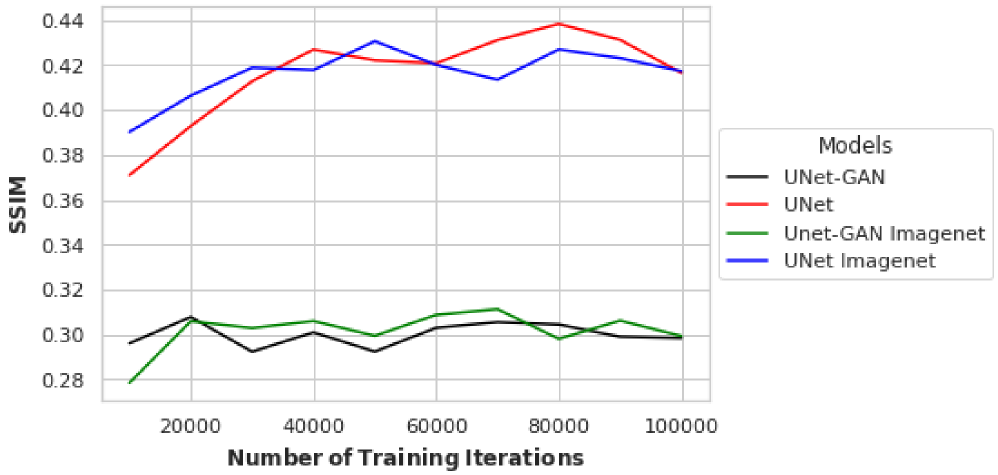

Fig 2. SSIM scores for four reconstruction pipelines.

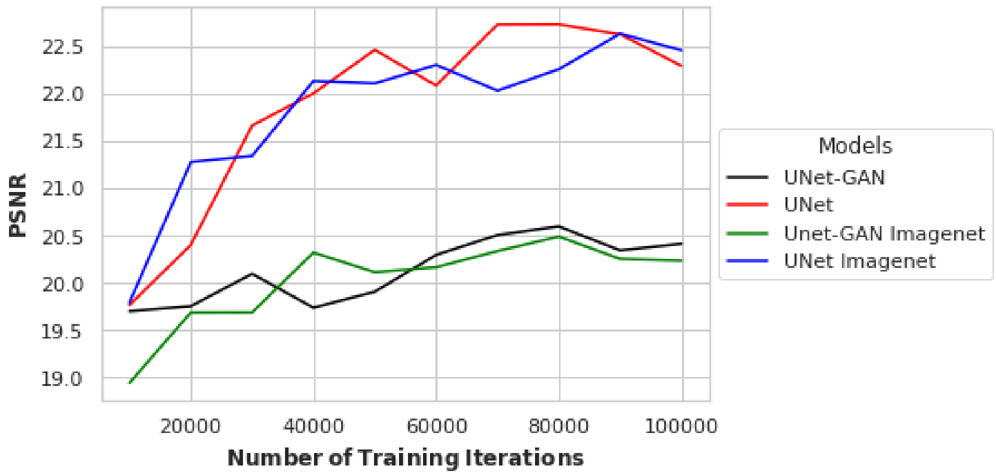

Fig 3. PSNR scores for four reconstruction pipelines.

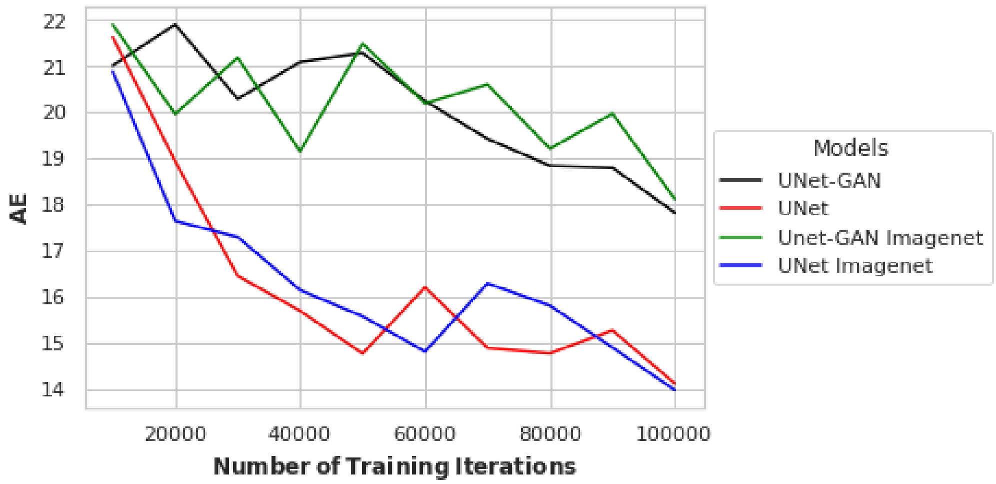

Fig 4. AE scores for four reconstruction pipelines.

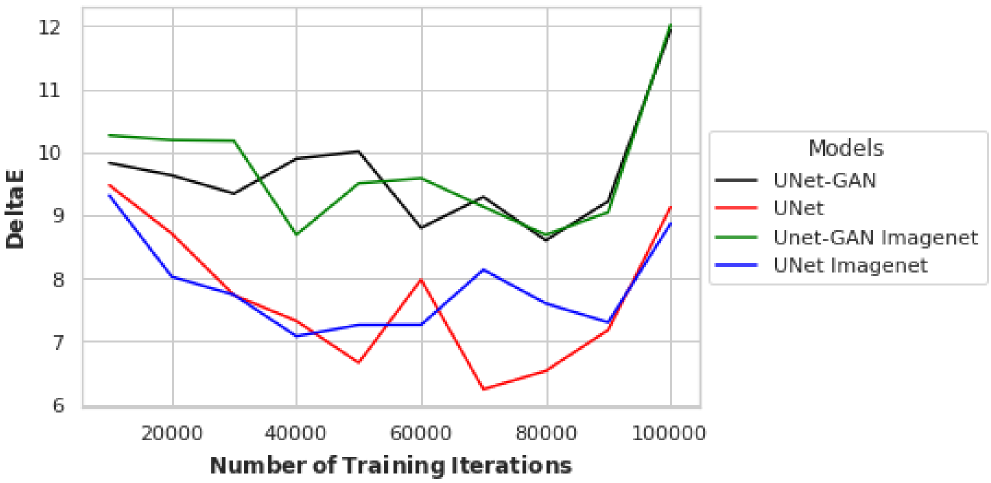

Fig 5. Delta E scores for four reconstruction pipelines.

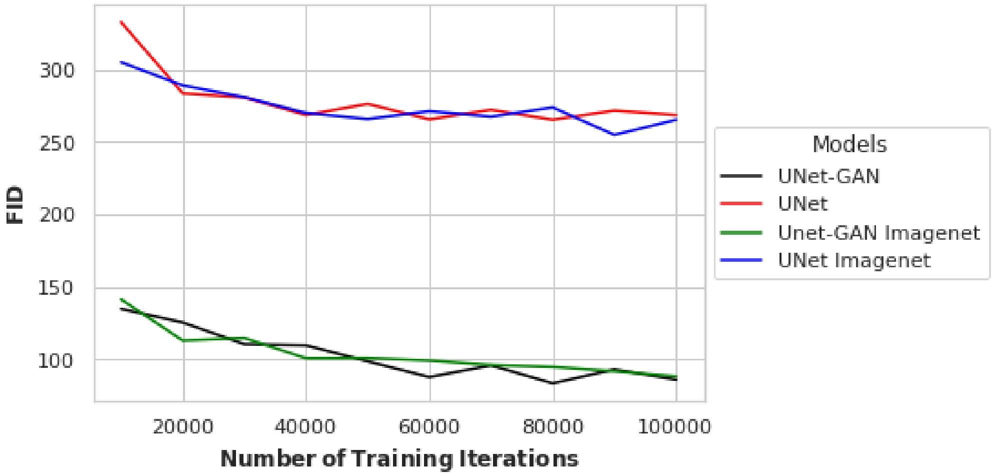

Fig 6. FID scores for four reconstruction pipelines.

ImegeNet weights did not significantly alter the models without pretraining. A possible explanation could be the difference in the domain of the ImageNet dataset, which does not contain a lot of human images, while human portraits predmonated the current study's dataset. Therefore, we elected to use the simpler training settings without including ImageNet pretrained weights. However, it is not clear which model, UNet or UNet-GAN performs better as the metrics gave very controversial results. Therefore, we visually inspected the patches of UNet and UNet-GAN and compared them with the ground truth (Fig. 7).

Fig. 7 demonstrates that UNet produced a blurry result, and the patch from UNet-GAN almost perfectly reconstructed the ground truth. The metric that was most correlated with our conclusions was FID, therefore, we used it as the major metric and reported it in the main paper. Combining everything together, we picked UNet-GAN without ImageNet weights and used FID as the guiding metric for quality of image reconstruction. Moreover, the minimum is reached when the model was trained up to 80K iteration.

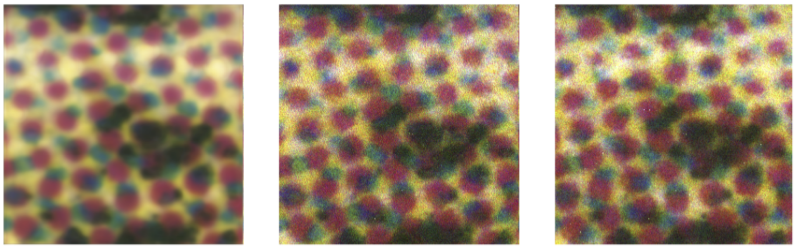

Fig 7. *(left)* Patch generated by the UNet architecture. *(middle)* Patch generated by the UNet architecture with adverserial loss. *(right)* Ground truth RGB patch.

Wavelength selection

For our experiments we had three infrared images with illumination wavelengths of 718, 777, 807 nm. To determine optimal visible wavelength image reconstruction using infrared inputs, we evaluated image reconstruction for all single wavelengths, their pairwise combinations and a combination of all three infrared wavelengths. The evaluation was performed using the validation dataset. We also wanted to verify that the best performing model was at 80K iterations.

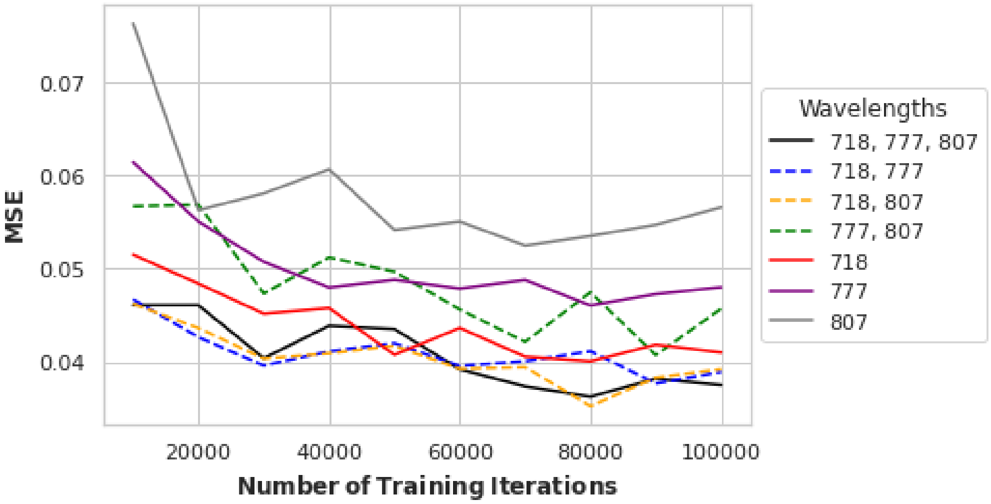

Fig 8. MSE scores for different wavelength combinations.

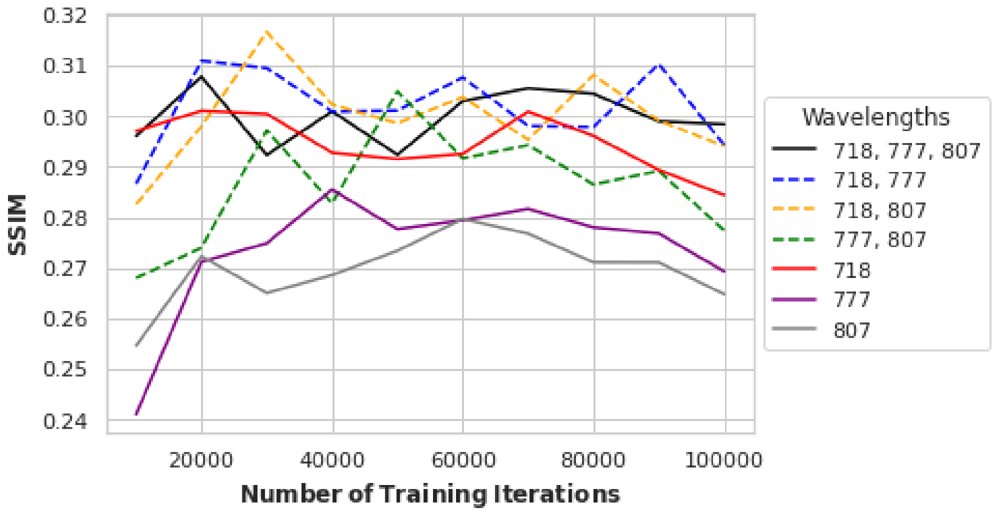

Fig 9. SSIM scores for different wavelength combinations.

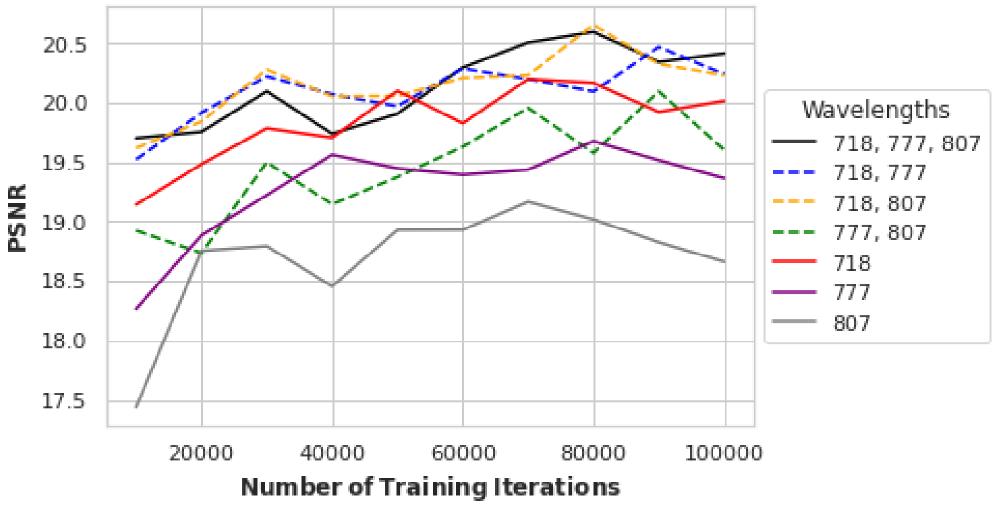

Fig 10. PSNR scores for different wavelength combinations.

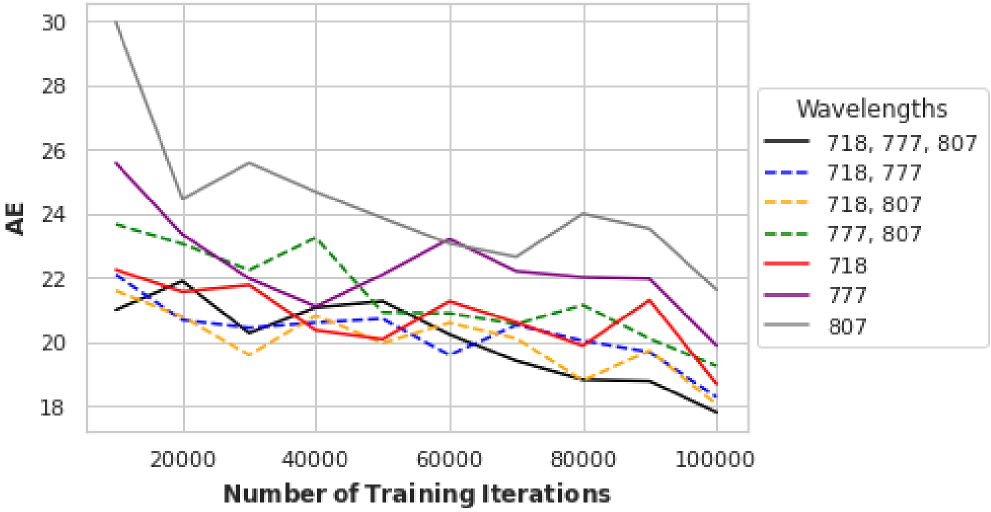

Fig 11. AE scores for different wavelength combinations.

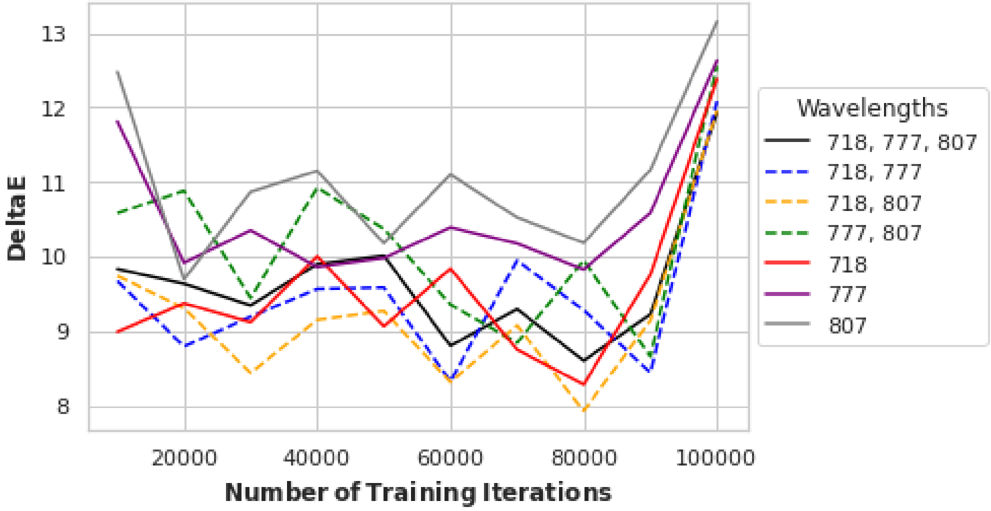

Fig 12. Delta E scores for different wavelength combinations.

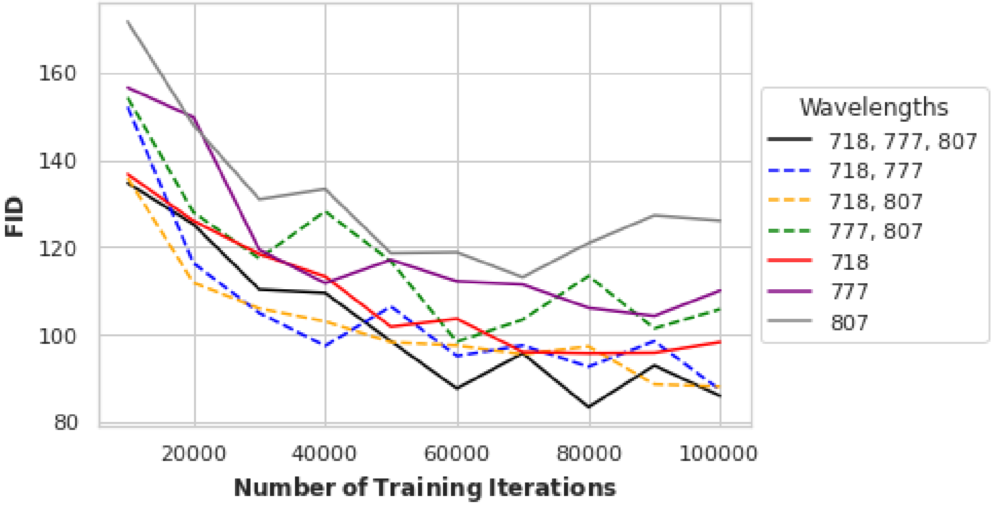

Fig 13. FID scores for different wavelength combinations.

FID identified that three wavelengths gave the best result when training occurred for 80K iterations. We used these parameters for our final evaluations on the test dataset and comparison to the baseline linear regression model.

Evaluation on the test dataset and comparison with the baseline

Our final step was evaluation on the test dataset and compare it with the baseline linear regression model. We picked our best model at 80K iteration, its counterpart without adversarial loss, i.e. UNet, again at 80K, and linear regression. The following Figures report the scores on all the metrics but we were focused only on FID.

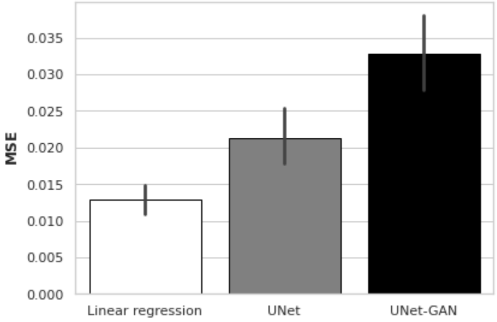

Fig 14. MSE scores for the baseline, UNet and UNet-GAN.

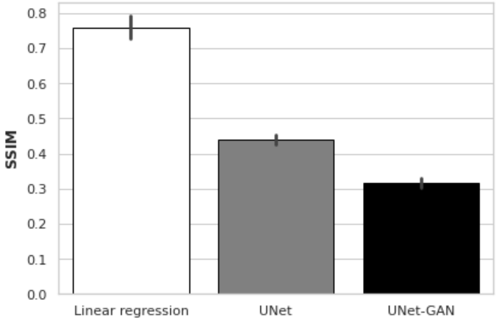

Fig 15. SSIM scores for the baseline, UNet and UNet-GAN.

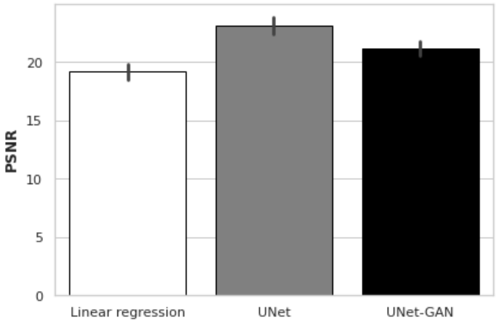

Fig 16. PSNR scores for the baseline, UNet and UNet-GAN.

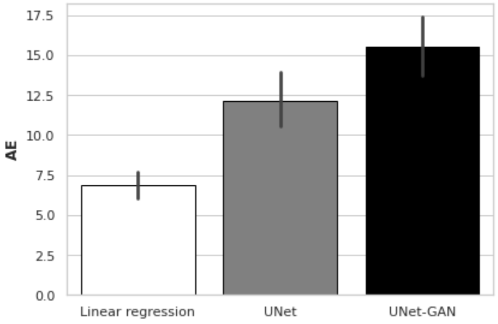

Fig 17. AE scores for the baseline, UNet and UNet-GAN.

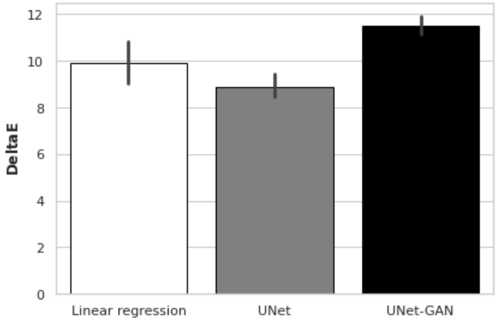

Fig 18. Delta E scores for the baseline, UNet and UNet-GAN.

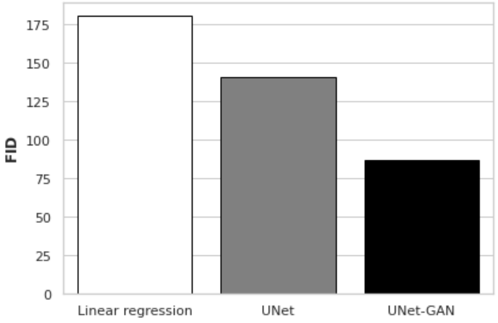

Fig 19. FID scores for the baseline, UNet and UNet-GAN.

To demonstrate model performance in relation to the quantitative results, Fig. 20 provides a representative example of trained model output when using three infrared wavelength inputs to predict the ground truth visible spectrum image. It is evident that the simple linear regression model produces images with color features not similar to the ground truth. In contrast, the deep architectures better captured the colors of the target RGB ground truth image. While the UNet and UNet-GAN reconstructions appear similar to each other when viewed as a gross image, the patch analysis shown in Fig. 7 demonstrates the superiority of the adversarial network which is also reflected by the lower FID score (Fig. 19).

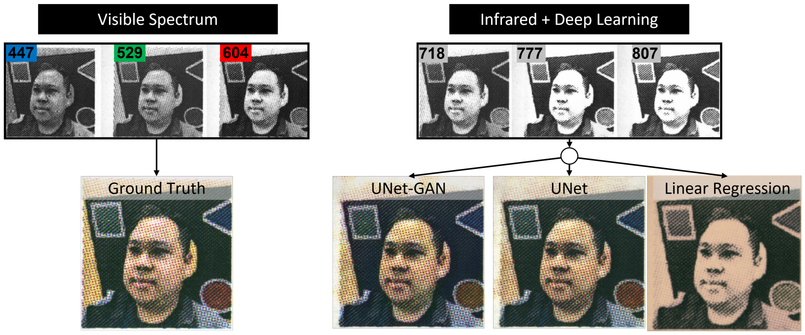

Fig 20. (*left*) Visible spectrum ground truth image composed of red, green and blue input channels. (*right*) Predicted reconstructions for UNet-GAN, UNet and linear regression using 3 infrared input images.

Fig. 21 shows arithmetic differences between ground truth and predicted images, which further solidifies the quality of our predictions. The Arithmetic difference was computed using imageJ's image calculator function to subtract an array of predicted images from an array of ground truth images to produce an array of images where difference between the two image sets is visualized.

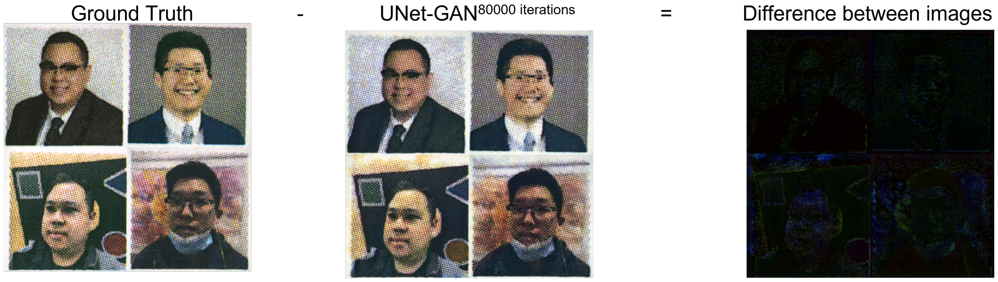

Fig 21. Arithmetic differences between ground truth and predicted images. The prevalence of dark colors, i.e. values close to zero, means that the predictions are very close to the ground truth.

Inference time

To decrease inference time, we tried varying the backbone of the generator and also explored different generator architectures. Table 1 shows the MSE scores and inference times for several combinations of architectures and backbones. It is evident than substituting VGG16 encoder with MobileNet did not significantly worsen the result, however, the inference time did not improve either. Although, FPN, PSPNet, and LinkNet improved (i.e decreased) the inference time, the quality of reconstructions (as measured by the MSE) also dropped.

Table 1. Comparison of different generator architectures.

Generator architecture	Backbone	Number of parameters (M)	MSE	Inference time (ms)
U-Net	VGG16	23.749	0.0078 ± 0.0010	356 ± 31
U-Net	MobileNet	6.629	0.0089 ± 0.0003	346 ± 16
FPN	MobileNet	4.216	0.0203 ± 0.0031	131 ± 10
PSPNet	MobileNet	2.273	0.0208 ± 0.0008	107 ± 1
LinkNet	MobileNet	4.320	0.0177 ± 0.0028	193 ± 22

Substituting VGG16 network for MobileNet did not significantly change MSE score nor inference time. However, the number of parameters decreased by almost a factor of 4. Although FPN, PSPNet, and LinkNet improved (i.e decreased) the inference time, the quality of reconstructions (as measured by the MSE) also dropped. Training and testing was performed on the Natural Images dataset and MSE scores and inference times evaluated for three held-out samples.

References

1. Heusel M, Ramsauer H, Unterthiner T, Nessler B, Klambauer G, Hochreiter S. GANs Trained by a Two Time-Scale Update Rule Converge to a Nash Equilibrium. CoRR. 2017;abs/1706.08500.

2. Isola P, Zhu JY, Zhou T, Efros AA. Image-to-Image Translation with Conditional Adversarial Networks; 2018.
